# Trait Anxiety and Economic Risk Avoidance Are Not Necessarily Associated: Evidence from the Framing Effect

**DOI:** 10.3389/fpsyg.2017.00092

**Published:** 2017-01-31

**Authors:** Ruolei Gu, Runguo Wu, Lucas S. Broster, Yang Jiang, Rui Xu, Qiwei Yang, Pengfei Xu, Yue-Jia Luo

**Affiliations:** ^1^CAS Key Laboratory of Behavioral Science, Institute of Psychology, Division of Social and Engineering Psychology, Chinese Academy of SciencesBeijing, China; ^2^School of Social and Political Science, University of EdinburghEdinburgh, UK; ^3^Department of Behavioral Science, University of Kentucky College of MedicineLexington, KY, USA; ^4^Institute of Basic Research in Clinical Medicine, China Academy of Chinese Medical SciencesBeijing, China; ^5^Sichuan Research Center of Applied Psychology and Collaborative Innovation Center of Sichuan for Elderly Care and Health, Chengdu Medical CollegeChengdu, China; ^6^Institute of Affective and Social Neuroscience, College of Psychology and Sociology, Shenzhen UniversityShenzhen, China; ^7^Center for Emotion and Brain, Shenzhen Institute of NeuroscienceShenzhen, China; ^8^Neuroimaging Center, University Medical Center Groningen, University of GroningenGroningen, Netherlands

**Keywords:** decision-making, framing effect, trait anxiety, depression, risk avoidance

## Abstract

According to previous literature, trait anxiety is related to the tendency to choose safety options during risk decision-making, that is, risk avoidance. In our opinion, anxious people’s risk preference might actually reflect their hypersensitivity to emotional information. To examine this hypothesis, a decision-making task that could elicit the framing effect was employed. The framing effect indicates that risk preference could be modulated by emotional messages contained in the description (i.e., frame) of options. The behavioral results have showed the classic framing effect. In addition, individual level of trait anxiety was positively correlated with the framing effect size. However, trait anxiety was not correlated with risk-avoidance ratio in any condition. Finally, the relationship between anxiety and the framing effect remained significant after the level of depression was also taken into account. The theoretical significance and the major limitations of this study are discussed.

## Introduction

Anxiety is a negative emotion characterized by anticipatory affective, cognitive and behavioral responses towards a possible threat (Grupe and Nitschke, 2013). The concept of anxiety is multifaceted including state anxiety and trait anxiety (Endler and Kocovski, 2001). State anxiety refers to a transient level of physiological arousal and feelings of vigilance, dread, and tension; on the other hand, trait anxiety reflects an individual’s disposition to experience anxiety-relevant feelings or thoughts or to show anxiety-related behaviors (Spielberger et al., 1983; Bekker et al., 2003). Both state and trait anxiety are linked with abnormal decision-making behavior. Eisenberg et al. (1995) first discovered that the participants with higher level of trait anxiety were overwhelmingly prone to choose safety options over risky options (i.e., risk avoidance). Later, Raghunathan and Pham (1999) found that the manipulation of increasing participants’ state anxiety level also resulted in a stronger tendency to avoid risk. The idea of linking anxiety with risk avoidance has been confirmed by many follow-up studies (e.g., Wray and Stone, 2005; Maner and Schmidt, 2006; Giorgetta et al., 2012; for reviews, see Hartley and Phelps, 2012; Paulus and Yu, 2012). Hartley and Phelps (2012) summarized previous findings and concluded that: “either heightened arousal to risky choice options or increased interoceptive awareness of arousal responses (or an interaction of the two) may lead anxious individuals to be more risk averse.”

Nevertheless, we would like to point out that “risk” is a broad term that across different domains and may have caused misunderstandings in the literature. Most notably, Schonberg et al. (2011) remind their readers to be aware of the “gap” between naturalistic risk (e.g., drug abuse and skydiving) and economic risk. From the perspective of evolutionary psychology, anxiety is an adaptive emotion that protects people from potential dangers by guiding the attention toward threat-relevant information (Stein and Bouwer, 1997; Nesse, 1999). Therefore, it is not surprising that anxiety is inherently associated with more conservative behavior when facing naturalistic risk. Regarding economic risk, however, previous findings are more heterogeneous (e.g., Mano, 1992; Hockey et al., 2000; Miu et al., 2008; Tang et al., 2012; Zhang et al., 2015). For instance, in a series of experiments, Mitte (2007) discovered that the relationship between anxiety and risk-avoidance is unstable, depending on the way of response format (verbal vs. numerical). In our opinion, during economic decision-making, anxiety influences risk preference by raising the sensitivity to negative emotion (see also Browning et al., 2015). More specifically, anxious people may not avoid the economic risk *per se*, but the anticipatory negative emotion associated with the possibility of a larger loss (Engelmann et al., 2015). That is to say, anxiety and economic risk avoidance are not necessarily connected.

In most decision-making tasks, the risky options are related to stronger emotional reactions (Loewenstein et al., 2001), thus it is very difficult to distinguish the role of risk and that of emotion. We suggest that investigating the framing effect could shed light on this issue. The term “framing effect” refers to a phenomenon that people are more likely to choose the option framed (e.g., worded) in an emotionally positive way, but are less likely to do so when the same option is framed in a negative way (Tversky and Kahneman, 1981). Positive or negative emotion generated by the “frame” elicits approach or avoidance behaviors, respectively (Fagley et al., 2010). The framing effect was first discovered in the naturalistic rather than the economic domain, that is, the Asian disease problem (Tversky and Kahneman, 1981). Peng et al. (2014) have found that high trait-anxious participants were more likely to avoid risk when dealing with the Asian disease problem. However, Peng et al. (2014) investigated the effect of the self-frame (i.e., subjectively constructing the information in an ambiguous situation). Therefore, their study is unsuitable to test the hypothesis that trait anxiety is associated with heightened sensitivity to external emotional information. In the context of economic decision-making, De Martino et al. (2006) found that people prefer choosing the safety option when it is framed as a potential gain compared to when it is framed as a potential loss. In our opinion, the paradigm developed by De Martino et al. (2006) provides an opportunity to modulate the emotional effect independent of economic risk.

In one of our recent studies, Xu et al. (2013) reported that the level of trait anxiety [measured by Spielberger’s Trait Anxiety Inventory (STAI-T); see Spielberger et al., 1983] was correlated with the framing effect size. Anxious people are more likely to choose the safety option when it is described as a gain, but less likely to do so when it is described as a loss, indicating that they are more susceptible to emotional information when making decisions. This idea is supported by brain-imaging findings that trait anxiety was correlated with activations of the amygdala, which is a key region in the emotional circuit (Xu et al., 2013). However, it remains unclear if anxiety level is consistently related to economic risk avoidance regardless of how the options are framed. In addition, the reliability of the findings of Xu et al. (2013) is harmed by its relatively small sample size (20 participants in total). Regarding the importance of reproducibility in psychological research (e.g., Open Science Collaboration, 2015), the experiment should be replicated in a larger sample.

Finally, the close relationship between anxiety and depression should be taken into account (Stavrakaki and Vargo, 1986). Depression is characterized by feeling of low mood, sadness, and loss of interest, and can be recognized as either a state or a cluster of symptoms (Zung, 1965; Rottenberg, 2005). Anxiety and depression overlap with each other in many aspects such as the component of negative affect (Lonigan et al., 1994; Joiner et al., 1996). Also, in the field of clinical psychology, anxiety and depressive disorders are highly comorbid (Domschke and Dannlowski, 2010). Therefore, it is recommended that the research on anxiety should control the effect of depression as a confounding variable (Beuke et al., 2003). Seeing that the current study investigates trait anxiety rather than state anxiety, we used Zung’s self-rating depression scale (SDS) to measure the depression level, because the SDS estimates depressive symptoms in a prolonged period (Zung, 1965). According to Xu et al. (2013), the SDS score was not significantly correlated with the framing effect size. However, considering the high correlation between anxiety and depression (*r* = 0.3∼0.7 in many studies, e.g., Knight et al., 1983; Bjelland et al., 2002; *r* = 0.53 in the sample of Xu et al., 2013), more rigorous statistical methods are needed to examine the potential influence of depression on the data.

This study employed a new sample to complete the task designed by De Martino et al. (2006), which could reliably elicit the classic framing effect during risk decision-making (Roiser et al., 2009; Xu et al., 2013). Individual levels of trait anxiety and depression were measured and entered into behavioral analyses. Our hypotheses were: (a) the level of trait anxiety would be positively correlated with the framing effect size, but not the tendency of risk avoidance; (b) taking depression into account would not affect the relationship between anxiety and the framing effect.

## Materials and Methods

### Participants

Sixty-nine students from Beijing Normal University participated in the study. A total of six participants were excluded from data analysis due to failure to complete all the questionnaires or participation discontinuation. As a result, the final sample consisted of data from 63 participants (34 female). Informed consents were obtained from all participants. The experimental protocol was approved by the local Ethics Committee at Beijing Normal University.

The Chinese version of STAI-T was used to assess the level of trait anxiety. The Chinese version of SDS was used to assess self-reported symptoms of depression. Both scales have demonstrated internal consistency, convergent validity, and discriminate validity (STAI-T: Spielberger et al., 1983; Shek, 1993; SDS: Zung et al., 1965; Shu, 1993).

### Procedure

Before the experiment, participants received the instruction about the formal task and were given 12 practice trials. They were also informed that their task performance (i.e., total points) would contribute to final payment. The relationship between point thresholds and corresponding participant payment was shown on a table, which indicated that the range of possible earning was 20–100 Chinese Yuan.

**FIGURE 1:**
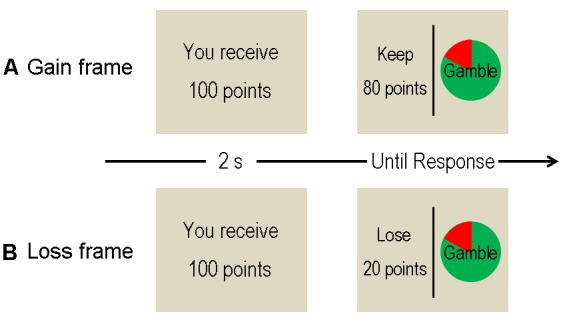
**Schematic diagram of a trial. (A)** The gain frame condition. **(B)** The loss frame condition.

**Figure 1** provides schematic illustration of a single trial. In the beginning of each trial, participants were shown a message screen (2 s) indicating an initial amount of reward (starting points: for instance, “You receive 100 points” in Chinese). There were four different starting point amounts (25, 50, 75, and 100 points), which were counterbalanced across conditions. However, participants would not actually get the reward before they make a decision between a “sure” and a “gamble” option, which appeared following the starting point presentation. The sure (safety) option indicates the amount of points that could be kept for certain if participants choose this option, while the gamble (risky) option indicates a win-or-nothing choice. Within each trial, the expected values of the sure and gamble options were identical and mathematically equivalent between conditions. The only difference between conditions was the description of the sure option; this option was described as money retained in the gain (positive) frame condition (e.g., “keep 80 points” of 100 points) but was described as money lost in the loss (negative) frame condition (e.g., “lose 20 points” of 100 points). In both conditions, the gamble option was presented identically as a pie chart depicting the probability of winning and losing in green and red color, respectively. There were four kinds of winning probabilities (20, 40, 60, and 80%) for the gamble option. Participants chose between the two options by pressing the F and J buttons on the keyboard (“F” for the option on the left side and “J” for the right side). The decision screen them disappeared immediately, which was followed by the next trial. No feedback was provided during the task (see De Martino et al., 2006; Xu et al., 2013, for more details). All experimental variables (including starting point amounts, positions of the two options, and winning probabilities) were fully counterbalanced between conditions.

The task was divided into two identical sessions comprised of 64 trials (32 gain frame and 32 loss frame; ordered pseudorandomly). At the end of the task, total earned points were displayed to participants. Stimulus display and behavioral data acquisition were conducted using E-Prime software 1.1 (Psychology Software Tools).

### Data Analysis

The framing effect in the current study was quantitatively defined as the difference between trials in which participants’ decisions were consistent with the frame (chose the sure option in the gain frame condition or the gamble option in the loss frame condition) and trials in which participants’ decisions ran counter to the frame (chose the gamble option in the gain frame condition or the sure option in the loss frame condition). Accordingly, the framing effect size was calculated as follows: (Gain_sure_ + Loss_gamble_) – (Gain_gamble_ + Loss_sure_) (De Martino et al., 2006).

Two-tailed one-sample *t*-test was used to examine the significance of the framing effect. Pearson correlation analysis (two-tailed) was used to determine the relationship between the framing effect and both anxiety and depression. Finally, linear regression analysis was performed to assess the unique contribution of anxiety.

For all the analyses, the results of descriptive statistics were reported as mean ± SD. The significance level was set at *p* = 0.05. In addition, the bias-corrected and accelerated (BCa) bootstrap 95% confidence intervals (CIs) were estimated based on bootstrapping with 5000 simulations (Efron, 1987). Statistical analysis was performed using IBM SPSS 19.0 (IBM Corporation).

## Results

### Self-Report Measures

In the whole sample, the STAI-T score was 38.25 ± 7.06 (range: 26–55) and the SDS score was 37.13 ± 9.37 (range: 23–54). The Pearson correlation (two-tailed) between two scales was significant (*r* = 0.292, *p* = 0.021, 95% CI [0.081,0.496]).

### Behavioral Results

The one-sample *t*-test revealed that the framing effect was significantly larger than zero (21.87 ± 20.20, *t*(62) = 8.594, *p* < 0.001); participants were more likely to make decisions in accordance with the frame (Gain_sure_ = 53.37%, Loss_gamble_ = 58.02%) rather than counter to the frame (Gain_gamble_ = 46.63%, Loss_sure_ = 41.98%).

**FIGURE 2:**
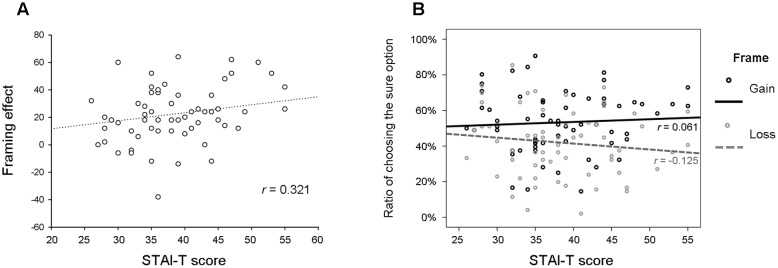
**(A)** A scatter plot of the correlation between STAI-T score and the framing effect size (*p* = 0.010). **(B)** A scatter plot of the correlations between STAI-T score and the ratio of choosing the sure option in the gain frame and the loss frame conditions (*p*s > 0.327).

Pearson correlation analysis revealed a positive correlation between the STAI-T score and the framing effect (*r* = 0.321, *p* = 0.010, 95% CI [0.095,0.520]) (**Figure 2A**). In contrast, the STAI-T score was not correlated with the risk-avoidance tendency (i.e., the ratio of choosing the sure option) in either the gain frame condition (*r* = 0.061, *p* = 0.637, 95% CI [–0.163,0.274]) or the loss frame condition (*r* = –0.125, *p* = 0.327, 95% CI [–0.347,0.107]) (**Figure 2B**). In addition, these effects were not sensitive to the amount of starting points or winning probabilities (results not showed for brevity). The SDS score was not significantly correlated with the framing effect (*r* = 0.212, *p* = 0.098, 95% CI [–0.005,0.414]), or the risk-avoidance tendency in the gain frame condition (*r* = –0.089, *p* = 0.492, 95% CI [–0.349,0.175]) or that in the loss frame condition (*r* = –0.199, *p* = 0.121, 95% CI [–0.428,0.041]).

Finally, a linear regression analysis was used to explain the framing effect based on the STAI-T and SDS scores (entered method). The regression model was significant [*F*(2,59) = 4.136, *p* = 0.021, *R*^2^ = 0.123]. Whereas the STAI-T score was a significant predictor of the framing effect (β = 0.805, *p* = 0.018, 95% CI [0.129,1.504]), the SDS score was not (β = 0.266, *p* = 0.309, 95% CI [–0.268, 0.752]).

## Discussion

Consistent with Xu et al. (2013), the current study has found a positive correlation between the level of trait anxiety (measured by STAI-T) and the framing effect size. That is, the influence of the description of the safety option on individual risk preference increased as a function of trait anxiety. These results indicate that people with high trait anxiety are more sensitive to the influence of contextual emotional information during risk decision-making. Consequently, their decisions are more likely to be in accord with the framing effect compared to those with low trait anxiety (Xu et al., 2013). In addition, the results of linear regression analysis have confirmed the independent role of trait anxiety after the depression factor (measured by SDS) was considered.

In contrast, the current study showed no evidence of the relationship between trait anxiety and economic risk avoidance, regardless of whether the safety option was framed as a potential gain or a potential loss. We suggest that trait anxiety and economic risk avoidance are not necessarily associated in certain circumstances, especially when incidental emotions generated by external information affect anxious people’s judgment. This idea is supported by one of our recent studies which discovered that during risk decision-making, the neural responses to feedback presentation were stronger under the influence of emotional facial expression in anxious participants compared to their non-anxious counterparts (Wang et al., 2016). As described in the Introduction, many studies have reported that the level of trait anxiety is consistently related to risk avoidance (Hartley and Phelps, 2012; Paulus and Yu, 2012). One should be very cautious if he/she would like to re-interpret the previous findings according to our theory, seeing that the current study has only examined one specific decision-making task. In our opinion, the key point of this study is that the relationship between trait anxiety and risk avoidance could be manipulated by the emotional context.

The possible mechanisms that connect trait anxiety with the framing effect should be discussed. At the physiological level, one of the major characteristics of anxiety is physiological hyperarousal (Joiner et al., 1996). It is possible that anxious individuals are more likely to be driven by emotional arousal during decision-making (Mano, 1992). As a result, their decision tends to be in accordance with the valence of emotional information, that is, making approach or avoidance responses under the influence of positive or negative emotion, respectively. At the brain level, trait anxiety magnitude is associated with structural and functional differences in the brain that may affect cognitive performance (Kuhn et al., 2011). Among the brain areas that are sensitive to trait anxiety level, the amygdala and the prefrontal cortex are most often highlighted (Kim and Whalen, 2009; Comte et al., 2015; Greening and Mitchell, 2015). According to Xu et al. (2013), trait anxiety level was positively correlated with amygdala-based “emotional” system activation when decisions were consistent with the framing effect, but negatively correlated with the anterior cingulate cortex (ACC)-based “analytic” system activation when decisions ran counter to the framing effect. It is thus possible that trait anxiety affects risk decision-making by modulating the activity of the brain networks being involved in the task. However, we should keep in mind that the major findings of this study were derived from correlation analyses. Strictly speaking, the direction of the causal relationship between trait anxiety and the framing effect remains undetermined.

The final part of this paper is about the limitations of the current study. First, regarding the high correlation between STAI-T and STAI-S scores (*r*≈0.8 in many studies; e.g., Wu et al., 2013), this study only collected STAI-T data. Seeing that trait anxiety and state anxiety are qualitatively distinct, follow-up research is necessary to examine the impact of state anxiety on the framing effect. Second, consistent with the task design in De Martino et al. (2006), only the description of the safety option, but not that of the risky option, was different between frame conditions. We did not change the original task because it reliably elicits the framing effect (see Introduction). Nevertheless, it would be interesting to check whether individual preference to the risky option would also be affected by the frame, and whether this effect would be sensitive to anxiety. Third, we predict that in naturalistic scenarios (e.g., the Asian disease problem), the relationship between anxiety and risk avoidance would not be overshadowed by the framing effect, but this idea was untested in the current study.

In a word, the current study indicates that the relationship between trait anxiety and risk decision-making is more complicated than what previous literature suggested, and further research is still needed to explore this issue.

## Ethics Statement

This study was carried out in accordance with the recommendations of American Psychological Association (2010) with written informed consent from all subjects. All subjects gave written informed consent in accordance with the Declaration of Helsinki. The protocol was approved by the local Ethics Committee at Beijing Normal University.

## Author Contributions

RG, RW, and PX conceived and designed the experiments. RW and PX performed the experiment. RG, YJ, and PX analyzed the data. RG, LB, YJ, and PX wrote the manuscript. RX, QY, and Y-JL contributed to manuscript revision.

## Conflict of Interest Statement

The authors declare that the research was conducted in the absence of any commercial or financial relationships that could be construed as a potential conflict of interest.
